# Supplementary material for: Engineering rules that minimize germline silencing of transgenes in simple extrachromosomal arrays in C. elegans
Source: Nat Commun. 2020 Dec 9;11:6300. doi: 10.1038/s41467-020-19898-0 (PMC7725773; doi:10.1038/s41467-020-19898-0)
Supplement: Supplementary file 1 — Supplementary Information [file 41467_2020_19898_MOESM1_ESM.pdf]

Supplementary Information

# Engineering rules that minimize germline silencing of transgenes in simple extra-chromosomal arrays in *C. elegans*.

Mohammed D. Aljohani[1,2], Sonia El Mouridi[1,2], Monika Priyadarshini[1,2], Amhed M. Vargas-Velazquez[1,2], and Christian Frøkjær-Jensen[1,3]

1. King Abdullah University of Science and Technology (KAUST), Biological and Environmental Science and Engineering Division (BESE), KAUST Environmental Epigenetics Program (KEEP), Thuwal, 23955-6900, Saudi Arabia.
2. These authors contributed equally: Mohammed D. Aljohani, Sonia El Mouridi, Monika Priyadarshini, Amhed M. Vargas-Velazquez.
3. Corresponding author: cfjensen@kaust.edu.sa

For more information please go to www.wormbuilder.org

Supplementary Information ........ 1
Supplementary Figures ........ 2
**Supplementary Figure 1**: Web interface to analyze periodic $A_n/T_n$ clusters (PATCs) ........ 2
**Supplementary Figure 2**: Germline expression from simple extra-chromosomal arrays is reproducible. ........ 3
**Supplementary Figure 3**: A broad range of promoters and 3' UTRs are permissive for germline expression from repressive chromatin. ........ 4
**Supplementary Figure 4**: A *C. briggsae* promoter and 3' UTR allow germline expression. ........ 5
briggsae ref[2] ........ 5
**Supplementary Figure 5**: Germline expression of a *ce-gfp* containing *smu-2* and artificial introns from simple arrays. ........ 6
Frøkjær-jensen et al reference[3] ........ 6
**Supplementary Figure 6**: Resistance to germline expression in the presence of PATCs and absence of plasmid backbone. ........ 7
**Supplementary Figure 7**: Co-injection of PATC-rich DNA does not increase germline expression and can result in aberrant germline morphology. ........ 8
**Supplementary Figure 8**: Germline expression from simple arrays from trans-spliced and non-transpliced promoters. ........ 9
**Supplementary Figure 9**: The effects of splicing and the plasmid backbone on germline expression. 10
**Supplementary Figure 10**: A backbone with nematode DNA is not required for germline expression from simple arrays. ........ 11
**Supplementary Figure 11**: Transgenes with the full endogenous *smu-1* coding sequence are silenced less and for a shorter time than fully synthetic *ce-gfp* transgenes. ........ 12
**Supplementary Figure 12**: Optimized Mos1 transposase concentration. ........ 13
Supplementary Methods ........ 14
Supplementary References ........ 16

Supplementary Figure 1

**a**

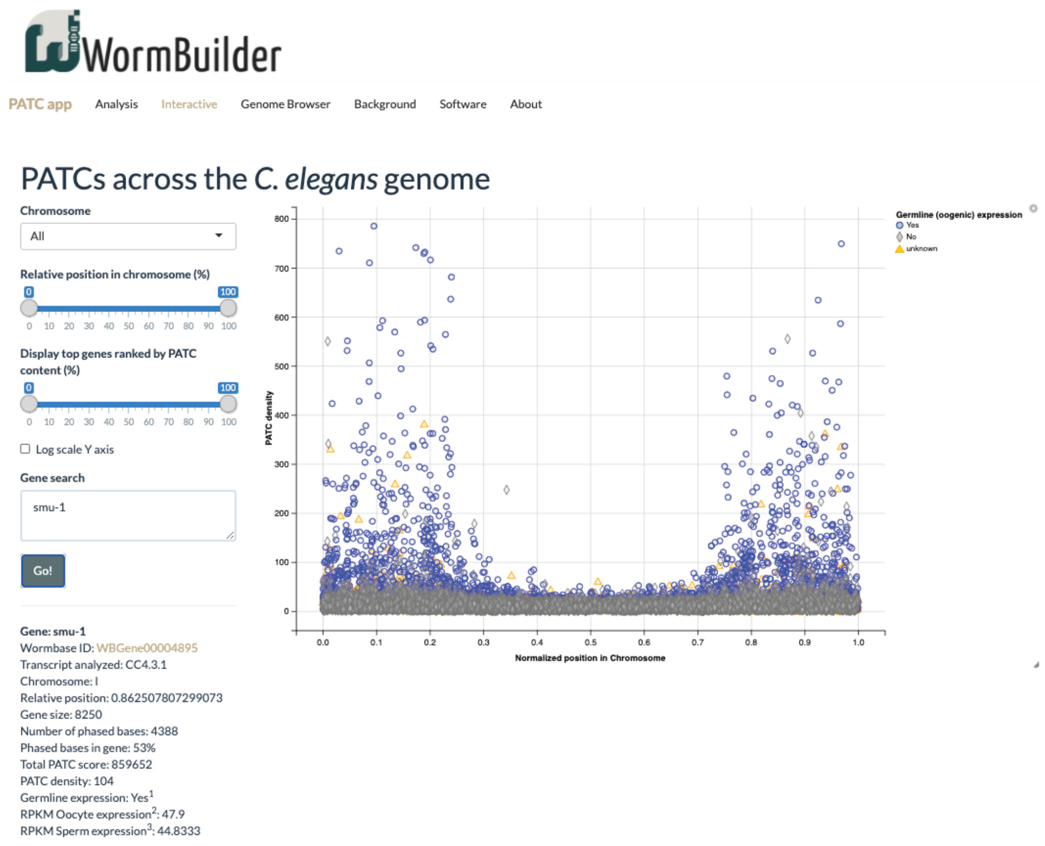


**b**

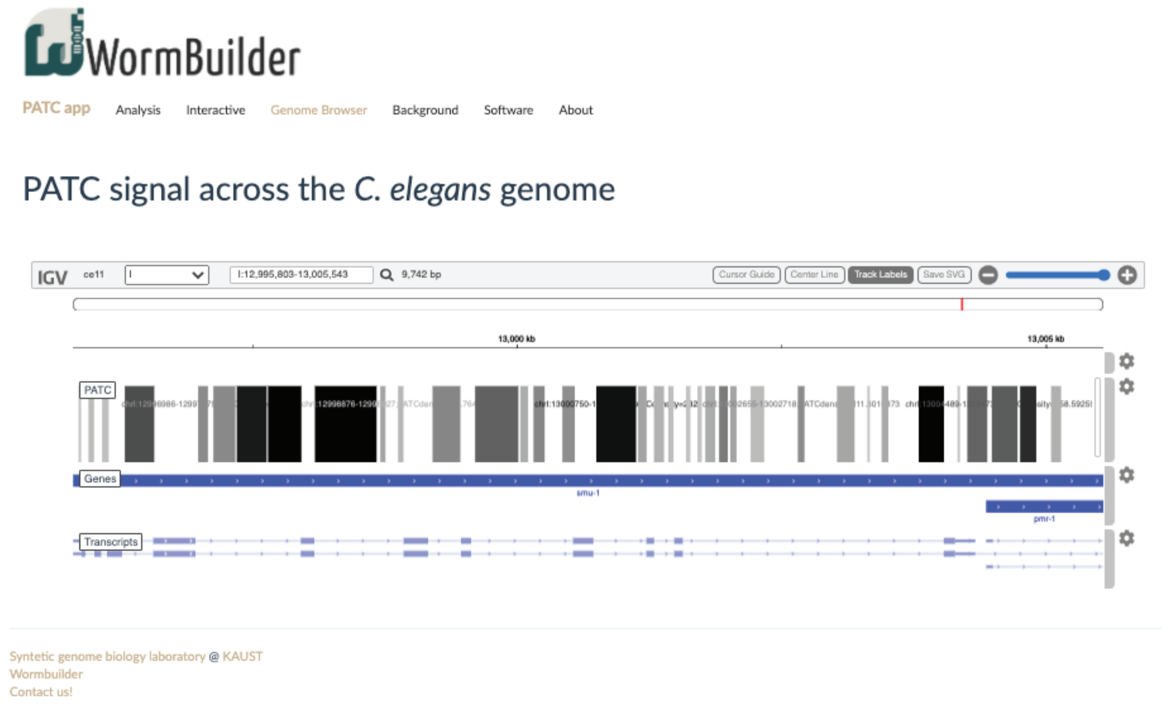


**Supplementary Figure 1**: Web interface to analyze periodic $A_n/T_n$ clusters (PATCs)
**a**. Screenshot of the interactive online database (www.wormbuilder.org/PATC/) where pre-calculated PATC values for all protein-coding *C. elegans* genes can be searched or visually browsed.
**b**. Gene browser track with PATC density as a track. The genome browser is implemented via the Integrated Genome Viewer (IGV)[1].

Supplementary Figure 2

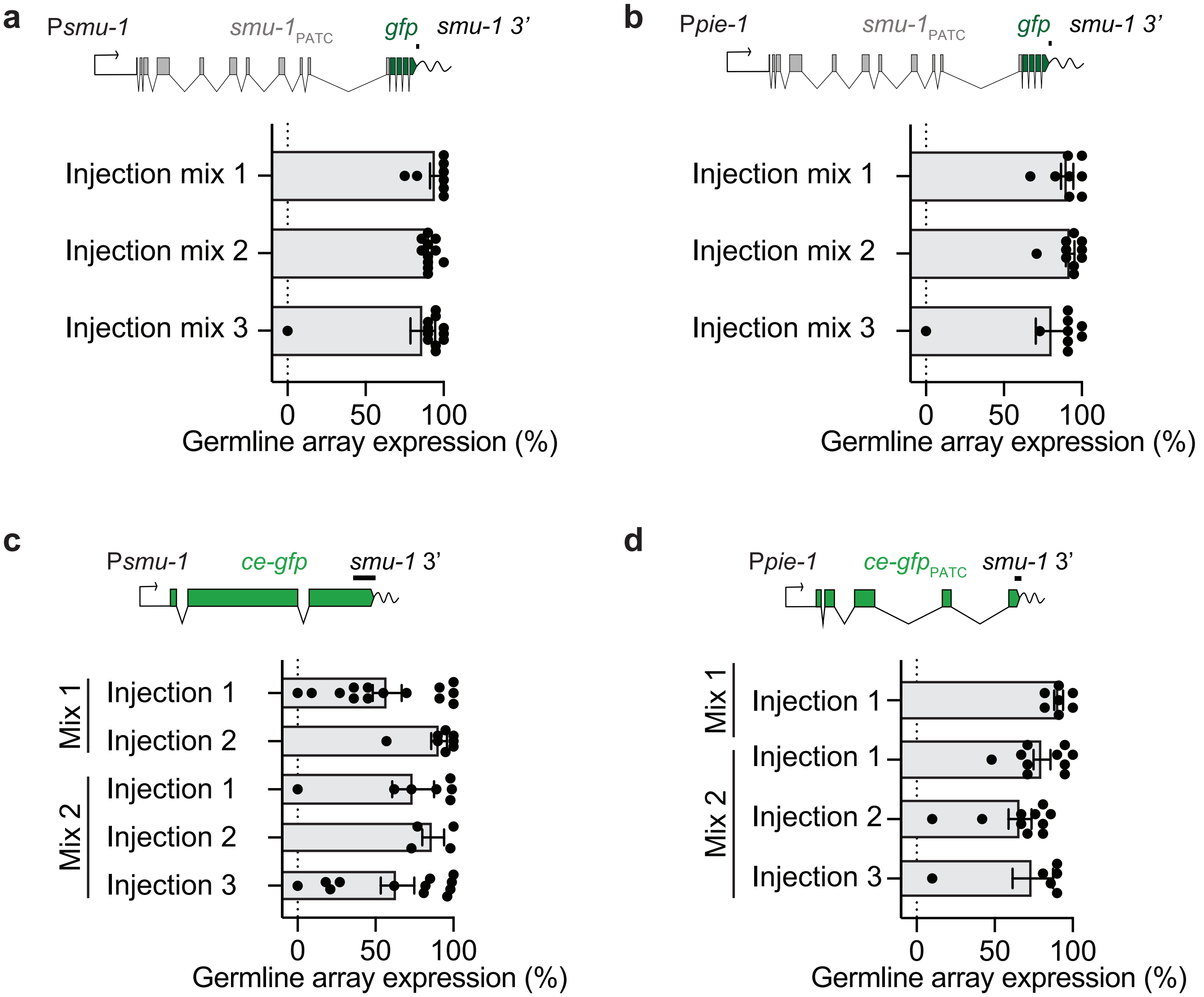


**Supplementary Figure 2**: Germline expression from simple extra-chromosomal arrays is reproducible. We repeated the same experiments (injection, strain propagation, and imaging) with several weeks or months in between. Sometimes, the same injection mix was re-used, but the injection mix was mostly remade (as indicated in individual figures). **a**. Three independent injections of a P*smu-1*::*smu-1*::*gfp* transgene. n = 8, 11, and 12 biologically independent transgenic lines (from top to bottom). **b**. Three independent injections of a P*pie-1*::*smu-1*:*gfp* transgene. n = 8, 10, and 9 biologically independent transgenic lines (from top to bottom). **c**. Five independent injections of a P*smu-1*::*ce-gfp* transgene with two injection mixes on different days. n = 14, 8, 7, 4, and 12 biologically independent transgenic lines (from top to bottom). **d**. Four independent injections of a P*pie-1*::*ce-gfp* transgene with two injection mixes on different days. n = 7, 10, 10, and 6 biologically independent transgenic lines (from top to bottom).

Fluorescence was quantified from transgenic animals carrying simple extra-chromosomal arrays imaged with a 40x or 63x oil objective at 25ºC. Each circle indicates one independent measurement of fluorescence scored from 11 animals from an independent transgenic line. Bars indicate the mean, and error bars indicate the SEM.

Supplementary Figure 3

**a**

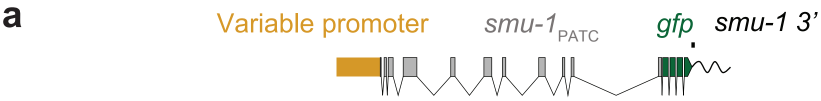


| Promoter | PATC Frequency | PATC Density | Length |
|---|---|---|---|
| P*smu-1* | 28% | 31 | 995 bp |
| P*smu-2* | 15% | 26 | 2071 bp |
| P*pie-1* | 47% | 86 | 3011 bp |
| P*pie-1*(short) | 61% | 97 | 1107 bp |
| (PATC) P*mex-5* | 58% | 100 | 1582 bp |

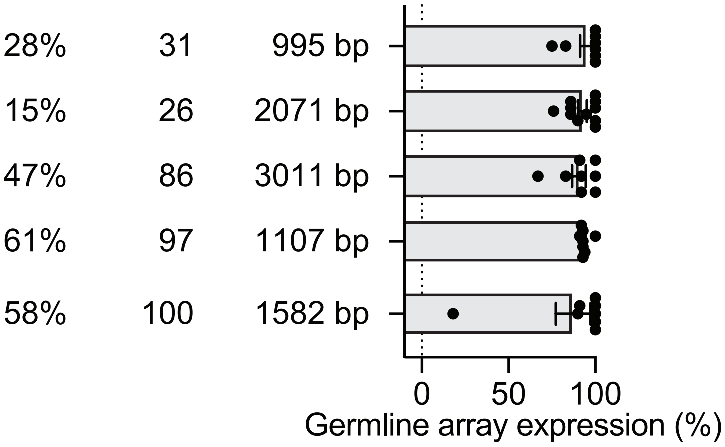


**b**

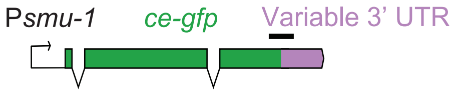


| 3' UTR | PATC Frequency | PATC Density | DNA length (bp) | UTR length (bp) |
|---|---|---|---|---|
| *smu-1* | 51% | 73 | 1023 | 194 |
| *smu-2* | 10% | 14 | 1436 | 66 |
| *pie-1* | 20% | 46 | 1051 | 94 |
| *tbb-2* | 0% | 15 | 332 | 155 |

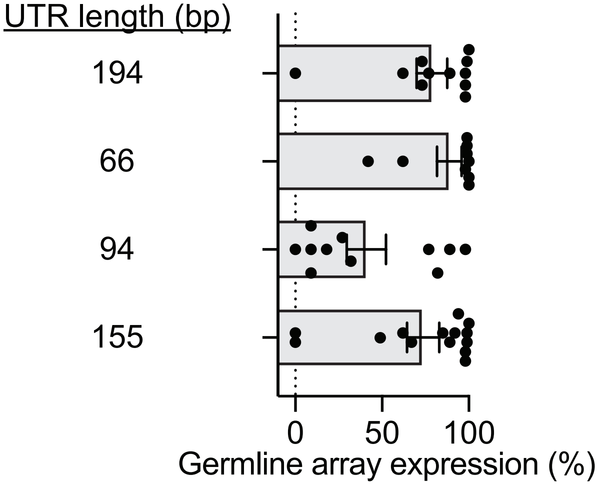


**Supplementary Figure 3:** A broad range of promoters and 3' UTRs are permissive for germline expression from repressive chromatin.
**a.** Top. Schematic of the transgene. Bottom. For all promoters, the PATC frequency and density are listed together with the promoter length. n = 8, 11, 8, 8, and 8 biologically independent transgenic lines (from top to bottom). **b.** Top. Schematic of the transgene. Bottom. For all 3' UTRs, the PATC frequency and density are listed. n = 11, 9, 11, and 14 biologically independent transgenic lines (from top to bottom).

Each data point is an average of 11 animals scored from each transgenic line. All animals were mounted and scored with a 63x oil objective at 25ºC. Bars indicate the mean, and error bars indicate the SEM.

Supplementary Figure 4

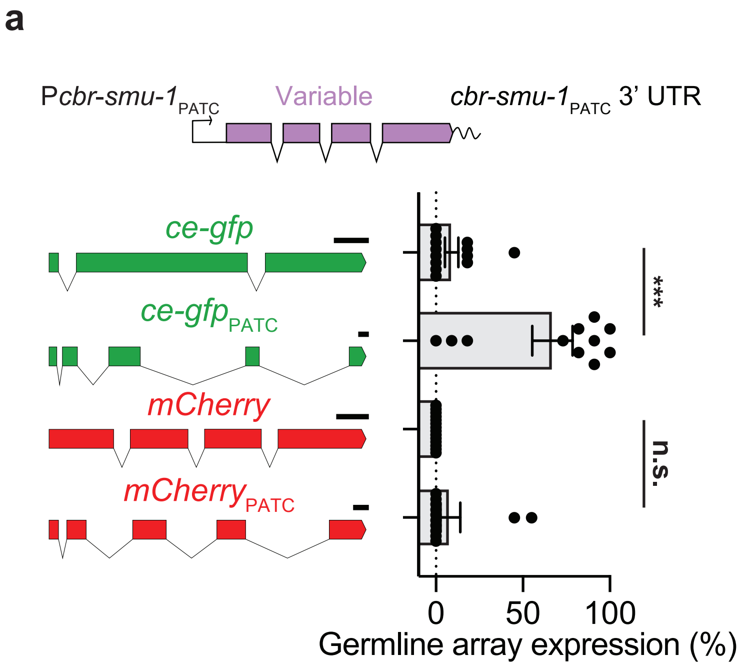


**Supplementary Figure 4**: A *C. briggsae* promoter and 3' UTR allow germline expression.
**a**. We tested the effect of using non-native promoters and 3' UTRs from a morphologically similar but evolutionarily divergent nematode species *C. briggsae*[2] using a codon-optimized *ce-gfp* and *mCherry*. n = 13, 11, 14, and 12 biologically independent transgenic lines (from top to bottom).

Each data point is the average of 11 animals scored from each transgenic line carrying extra-chromosomal arrays. All animals were mounted and scored for germline expression with 40x-60x oil objectives at 25ºC. Bars indicate the mean, and error bars indicate the SEM. Statistics: Two-tailed Mann-Whitney test *** p = 0.0006.

Supplementary Figure 5

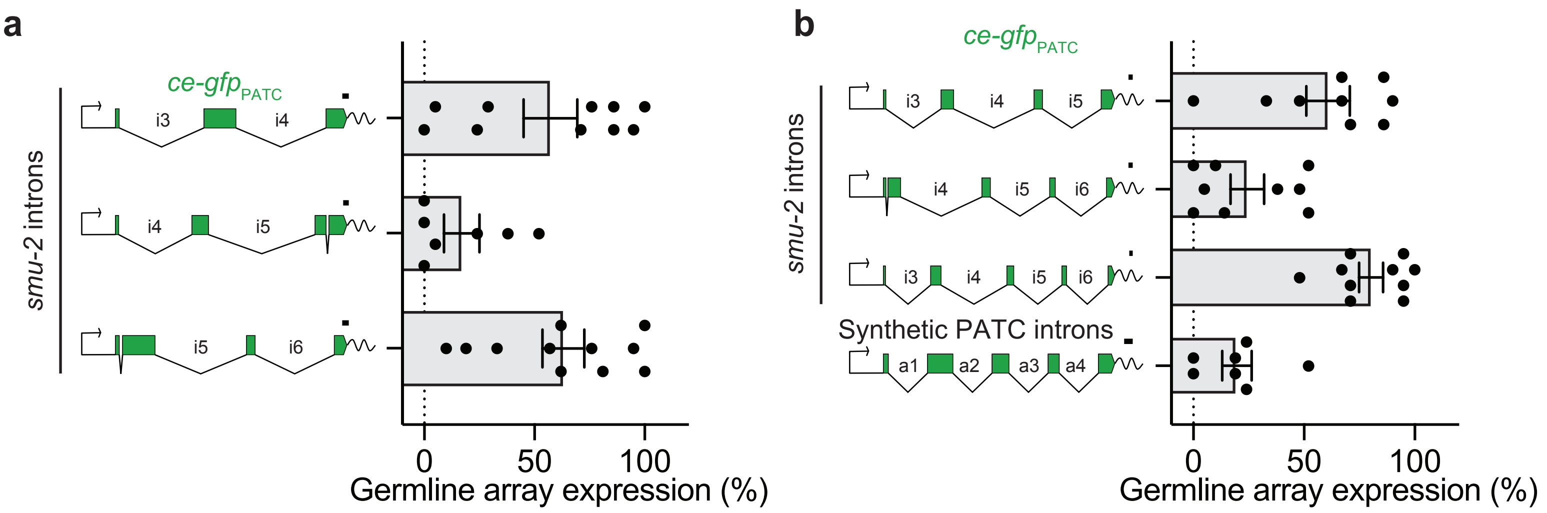


**Supplementary Figure 5**: Germline expression of a *ce-gfp* containing *smu-2* and artificial introns from simple arrays.

**a**. Germline fluorescence from P*pie-1*::*ce-gfp*$_{PATC}$ transgenes containing two *smu-2* introns from arrays. n = 10, 7, and 11 biologically independent transgenic lines (from top to bottom). **b**. Germline fluorescence from P*pie-1*::*ce-gfp*$_{PATC}$ transgenes containing three *smu-2* or artificial PATC-rich introns from arrays. n = 9, 9, 10, and 7 biologically independent transgenic lines (from top to bottom). Artificial introns were previously shown to improve germline expression from single-copy insertions[3].

Each data point is an average of 11 animals scored from each transgenic line. All animals were mounted and scored with 40x-63x oil objectives at 25ºC. Bars indicate the mean, and error bars indicate the SEM.

Supplementary Figure 6

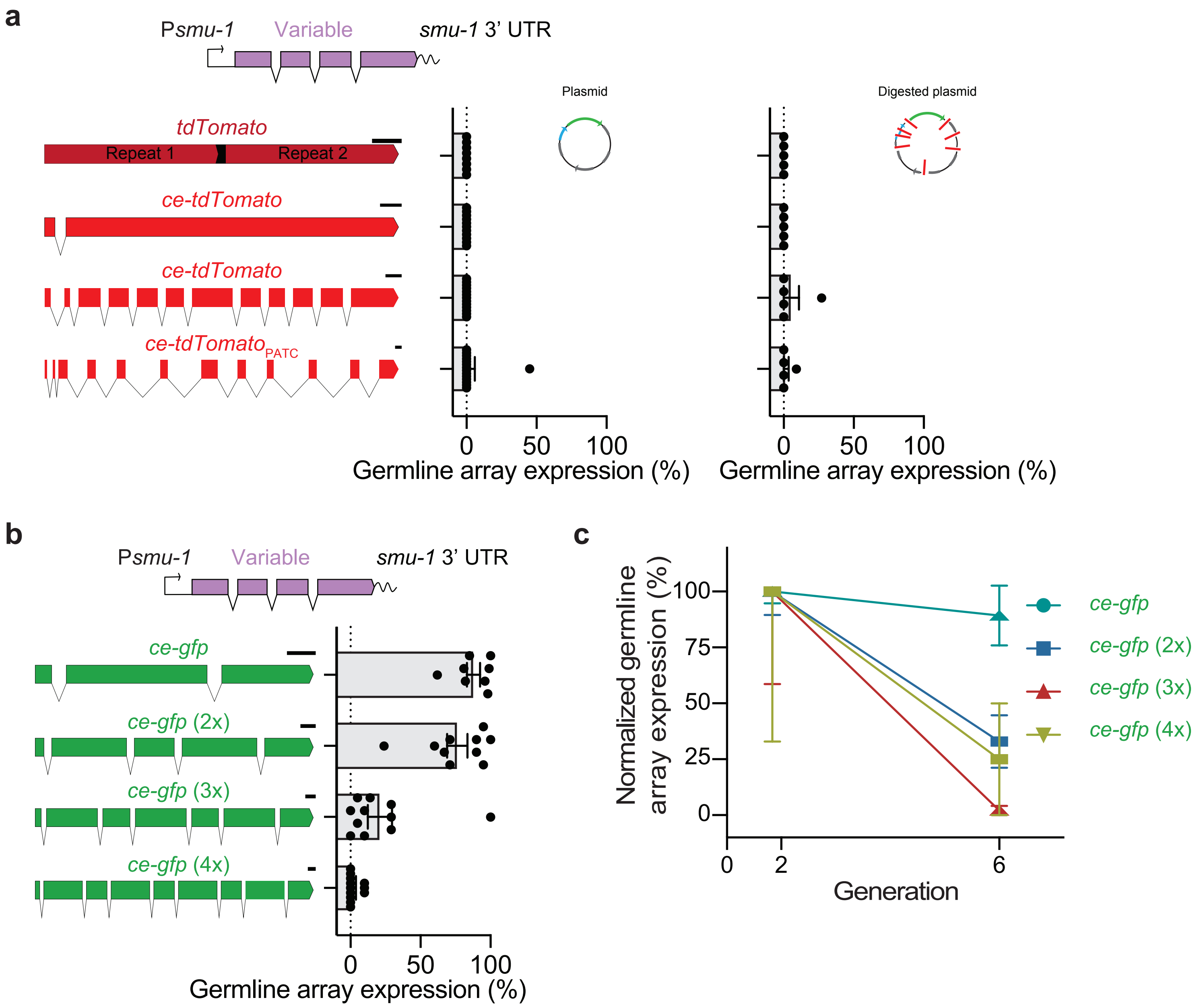


**Supplementary Figure 6**: Resistance to germline expression in the presence of PATCs and absence of plasmid backbone.

The effect of transgene structure, in particular tandem repeat structures, was tested using tandem-dimer tomato (*tdTomato*)[4] and *ce-gfp* tandem repeats. **a**. Quantification of germline expression from simple arrays of *tdTomato* and codon-optimized *tdTomato* (*ce-tdTomato*) in the germline under the P*smu-1* promoter and 3' UTR. Left: simple arrays with the injection of circular plasmids. Right: simple arrays with the injection of linear plasmids with the backbone digested with KpnI, EcoRV, and ApaLI. n = 8, 11, 13, and 15 biologically independent transgenic lines (undigested, from top to bottom). n = 4 biologically independent transgenic lines for all digested plasmids (right). **b**. Quantification of germline expression from simple arrays of transgenes with a variable number of direct repeats of *ce-gfp* fused together by flexible linker sequences under the *smu-1* promoter and 3' UTR. n = 8, 10, 11, and 12 biologically independent transgenic lines (from top to bottom). **c**. Perdurance of germline expression was quantified for the various *ce-gfp* transgenes two and six generations after establishing simple extra-chromosomal array lines. n = 8, 10, 11, and 12 biologically independent transgenic lines (top to bottom, legend). The average level of fluorescence was normalized to the starting levels of expression in the second generation.

Each data point is an average of 11 animals scored from each transgenic line carrying extra-chromosomal arrays. All animals were mounted and scored for germline expression with 40x-63x oil objectives at 25ºC. Bars indicate the mean, and error bars indicate the SEM.

Supplementary Figure 7

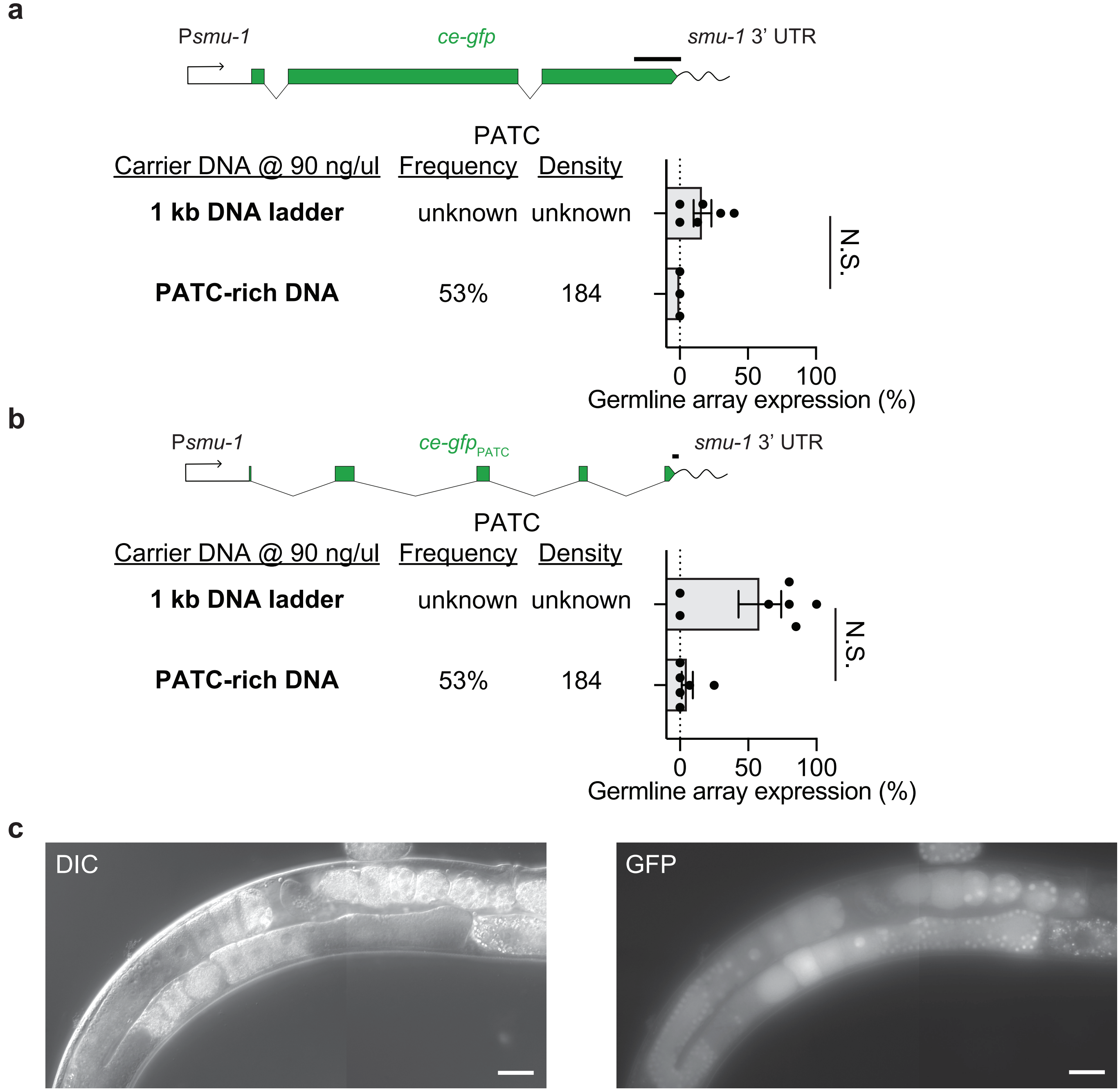


**Supplementary Figure 7**: Co-injection of PATC-rich DNA does not increase germline expression and can result in aberrant germline morphology.
**a**. Germline expression of a P*smu-1*::*ce-gfp* transgene co-injected with 90 ng/ul of 1 kb DNA ladder (top) or PATC-rich (bottom) stuffer DNA (a non-functional P*pie-1*::*smu-2*::*smu-1* UTR transgene). n = 6 and 3 biologically independent transgenic lines (top to bottom) **b**. Germline expression of a PATC-rich P*smu-1*::*ce-gfp* transgene co-injected with 90 ng/ul of 1 kb DNA ladder (top) or PATC-rich (bottom) stuffer DNA. n = 7 and 6 biologically independent transgenic lines (top to bottom) **c**. Image of an aberrant germline that is infrequently observed after injection with PATC-rich stuffer DNA. The animals have what appear to be two pachytene to oocyte transitions within the same gonad arm. This aberrant morphology was not verified in independent biological replicates. Scale bar = 20 micron.

Each data point is an average of 11 animals scored from each transgenic line. All animals were mounted and scored with 40x-63x oil objectives at 25ºC. Bars indicate the mean, and error bars indicate the SEM. Statistics: Two-tailed Mann-Whitney non-parametric test.

Supplementary Figure 8

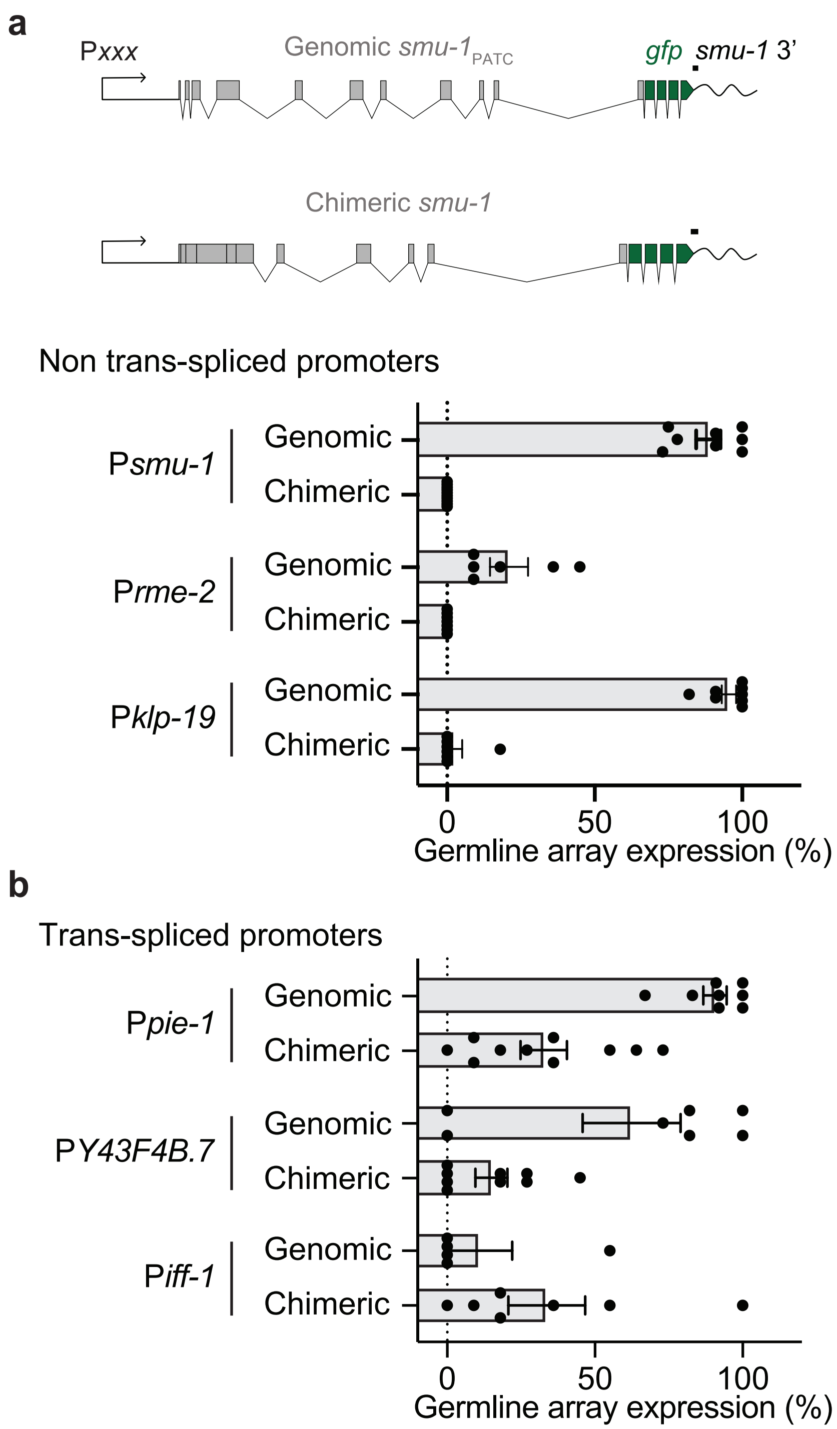


**Supplementary Figure 8**: Germline expression from simple arrays from trans-spliced and non-transpliced promoters.
We tested germline expression of transgenes with a full genomic *smu-1* coding region or a chimeric *smu-1* gene lacking the first five introns using various promoters. **a**. Germline expression from three promoters (P*smu-1*, P*rme-2*, and P*klp-19*) that are not SL1 or SL2 trans-spliced. n = 8, 10, 6, 7, 8, and 7 biologically independent transgenic lines (top to bottom). **b**. Germline expression from three SL1 trans-spliced promoters (P*pie-1*, P*Y43F4B.7*, and P*iff-1*). n = 8, 10, 7, 9, 5, and 7 biologically independent transgenic lines (top to bottom).

All germline fluorescence was quantified from transgenic animals carrying simple extra-chromosomal arrays imaged with a 40x or 63x oil objective at 25ºC. Each circle indicates one independent measurement of germline fluorescence scored from 11 animals from an independent transgenic line. Bars indicate the mean, and error bars indicate the SEM.

# Supplementary Figure 9

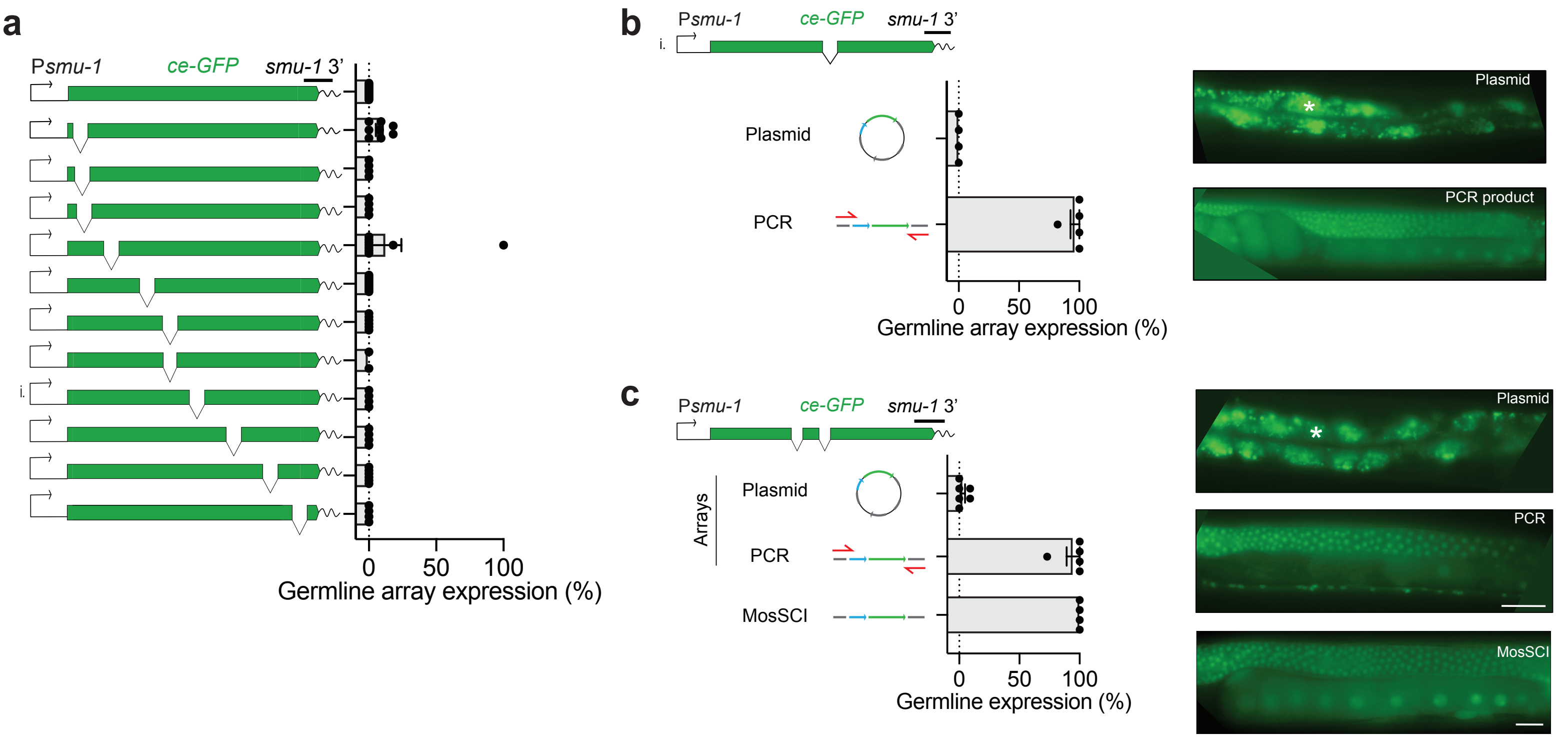


**Supplementary Figure 9**: The effects of splicing and the plasmid backbone on germline expression. **a**. Simple array germline expression of a P*smu-1*::*ce-gfp* transgene containing no synthetic introns (top) or a single synthetic intron along the gene body. n = 15, 8, 4, 4, 9, 9, 6, 2, 4, 4, 6, and 4 biologically independent transgenic lines (top to bottom). **b**.**c**. The effect of transgene context on the expression of *ce-gfp* containing two synthetic introns using the *smu-1* promoter. Images (right) show typical GFP expression. b. n = 4 and 5 biologically independent transgenic lines (top to bottom). c. n = 6, 5, and 4 biologically independent transgenic lines (top to bottom). Scale bar = 20 um. * = non-specific gut granule fluorescence. Conditions: Plasmid = simple arrays with circular plasmid at 25 ng/ul. MosSCI = single-copy transgene insertion at *ttTi5605* (Chr. II). PCR = simple arrays with PCR amplified transgene (no plasmid backbone).

All germline fluorescence was quantified from transgenic animals imaged with a 40x or 63x oil objective at 25ºC. Each circle indicates one independent measurement of germline fluorescence scored from 11 animals from an independent transgenic line. Bars indicate the mean, and error bars indicate the SEM.

Supplementary Figure 10

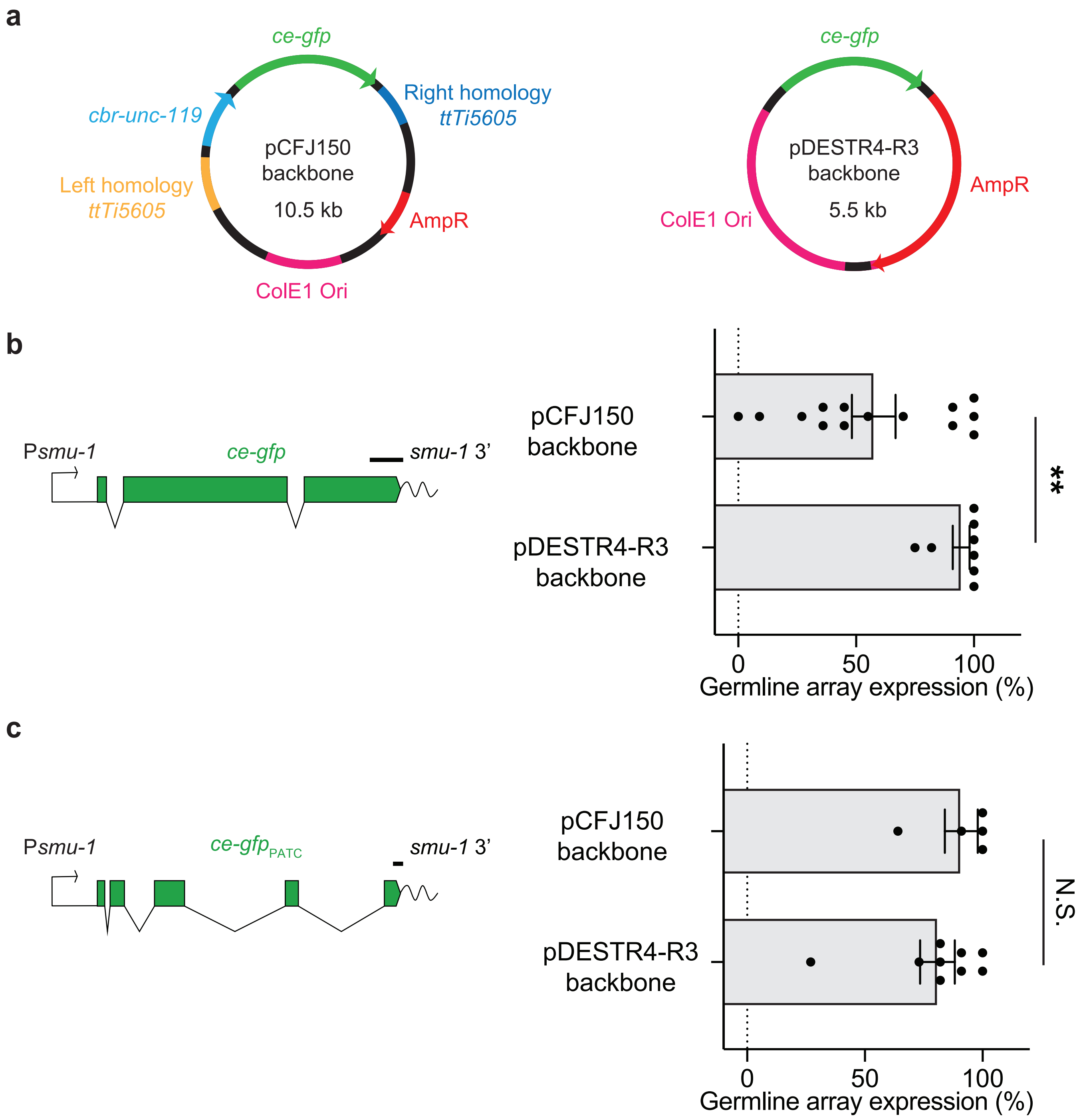


**Supplementary Figure 10**: A backbone with nematode DNA is not required for germline expression from simple arrays

**a**. Schematic overview of the mosSCI vector pCFJ150 with a *cbr-unc-119* and two 1.5 kb *C. elegans* homology regions ("left" and "right" homology to *ttTi5605*) flanking the ce-gfp transgene and the standard pDESTR4-R3 vector used for Three-Fragment multi-site Gateway reactions (ThermoFisher). **b**. Germline fluorescence from arrays formed from injection with a *ce-gfp* cloned into either pCFJ150 or pDESTR4-R3. n = 14 and 8 biologically independent transgenic lines (top to bottom). **c**. Germline fluorescence from arrays formed from injection with a *ce-gfp*$_{PATC}$ cloned into either pCFJ150 or pDESTR4-R3. n = 5 and 9 biologically independent transgenic lines (top to bottom).

Each data point is an average of 11 animals scored from each transgenic line. All animals were mounted and scored with 40x-63x oil objectives at 25ºC. Bars indicate the mean, and error bars indicate the SEM. Statistics: Two-tailed Mann-Whitney non-parametric test. ** p < 0.01.

Supplementary Figure 11

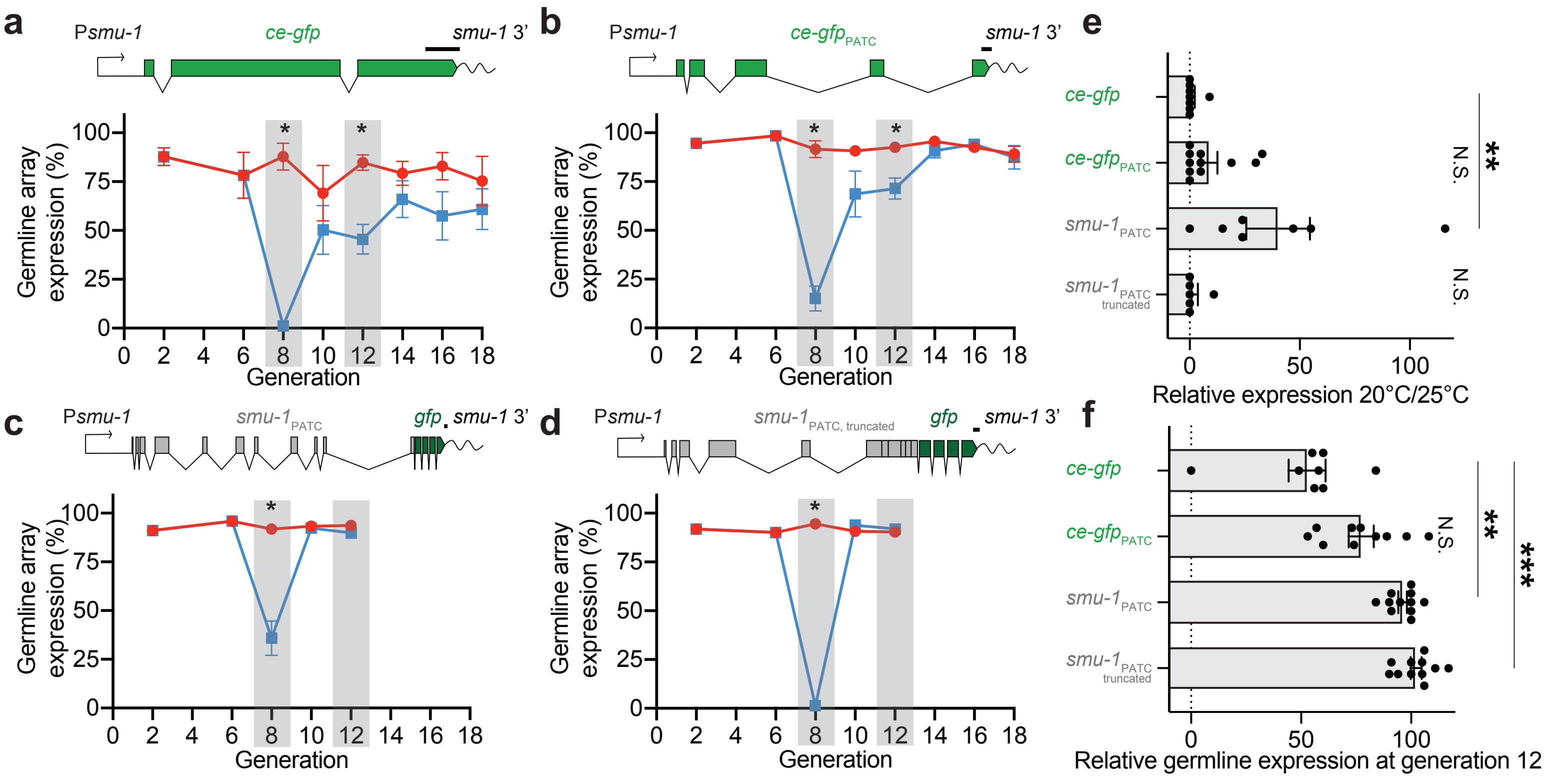


**Supplementary Figure 11**: Transgenes with the full endogenous *smu-1* coding sequence are silenced less and for a shorter time than fully synthetic *ce-gfp* transgenes.
The data is repeated from Figure 5 and analyzed at generation 8 and 12 for silencing. The GFP fluorescence from extra-chromosomal arrays in the germline was quantified over several generations. Animals were grown for two generations at a lower temperature (20ºC) (blue line) and shifted back to 25ºC. Control animals (red line) were propagated continuously at 25ºC.
**a**. Germline expression of *ce-gfp* containing no PATCs under the PATC-rich *smu-1* promoter and 3' UTR. n = 8 biologically independent transgenic lines. **b**. Germline expression of *ce-gfp* with PATC-rich sequences in the promoter, introns, and 3' UTR. n = 11 biologically independent transgenic lines. **c**. Germline expression of a *gfp*-tagged "full" length P*smu-1*::*smu-1* transgene. n = 11 biologically independent transgenic lines. **d**. Germline expression of a *gfp*-tagged chimeric P*smu-1*::*smu-1* transgene with the last five introns removed. n = 11 biologically independent transgenic lines. **e**. A plot of the relative silencing after two generations at 20ºC for the four transgenes. n = 8, 11, 7, and 6 biologically independent transgenic lines (top to bottom). **f**. A plot of the relative silencing four generations after returning animals to 25ºC for all four transgenes. n = 8, 10, 11, and 11 biologically independent transgenic lines (top to bottom)

All germline fluorescence was quantified from transgenic animals carrying simple extra-chromosomal arrays imaged with a 40x or 63x oil objective at the indicated temperatures. Each circle indicates one independent measurement of germline fluorescence scored from 11 animals from an independent transgenic line. Bars indicate the mean, and error bars indicate the SEM.
Statistics: (e,f) Kruskal-Wallis one-way ANOVA. Multiple comparisons: Dunn's test. (e)** p = 0.028 (f) ** p = 0.0013, *** p<0.0001.

Supplementary Figure 12

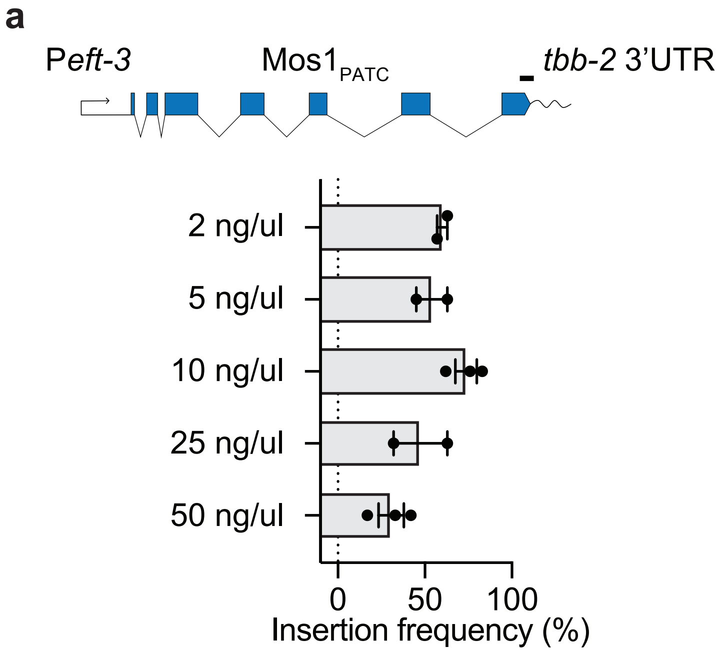


**Supplementary Figure 12**: Optimized Mos1 transposase concentration.
**a**. We tested the effect of concentration on generating single-copy MosSCI insertions using a codon-optimized and PATC-enriched Mos1 transpose transgene ($Mos1_{PATC}$). Insertion frequency of a 4.7 kb P*eft-3*:*gfp* transgene at *oxTi365* (Chr. V). n = 2, 2, 3, 2, and 3 biologically independent injections. Bars indicate the mean, and error bars indicate the SEM.

## Supplementary Methods

### Molecular biology

Molecular biology was performed using standard protocols and commercial available reagents. Molecular biology followed standard protocols, primarily using three-fragment Gateway Cloning (Life Sciences), Gibson Assembly[5,6], Golden Gate cloning[7], oligos and Gblocks from IDT (Idaho), gel purification kits from Zymo Research (#D4002), T7 (L6020L), and T4 DNA (L603-HC-L) ligases from Enzymatics. All other enzymes were purchased from New England Biolabs.

Transgenes were codon-adapted for high expression[8], depleted of piRNA homology by requiring at least three mismatches to published piRNAs[9–11], and PATC-rich introns were inserted by Golden-Gate assembly[7]. We used a dual nuclear localization signal with SV40 added to the N-terminus, and an *egl-13* NLS added to the C-terminus[12].

We generated most plasmids by three-fragment multisite Gateway reactions (ThermoFisher Cat. no. 12538200). All polymerase chain reactions were done using a high fidelity Phusion polymerase (New England Biolabs, cat. no. F530S). Entry vectors were generated by BP reactions and validated by Sanger sequencing. Expression vectors were generated by three-fragment LR reactions and verified by restriction digest. All reactions were designed using the free molecular biology editor "A plasmid Editor" (ApE) developed and maintained by M Wayne Davis. Plasmid names for each figure are listed in the Source Data file, and annotated DNA sequences in GenBank format are included in Supplementary Data 1. All plasmids are available upon request from Addgene or from the corresponding author.

A step-by-step protocol describing the Golden-Gate-based method for inserting PATC-rich introns into a synthetic transgene can be found at Protocol Exchange[13]. Briefly, we inserted PATC-rich introns into synthetic transgenes containing one to four short, synthetic introns compatible with Golden-Gate-based cloning using BsaI[14]. We mixed 150 ng of the synthetic transgene with equimolar amounts of donor plasmids containing the appropriate, compatible PATC-rich introns from Table 1. We performed reactions with BsaI-HF and T4 ligase in a 15 ul volume and 50x restriction digest and ligation cycles (5 min at 37°C and 5 min at 16°C). Following the Golden-Gate reaction, we removed potential unreacted (background) plasmids by incubating the mix with recBCD and BsaI-HF restriction enzyme for 30 minutes at 37°C and purifying the reaction mix with a gel purification column (Zymo Research). We transformed Top10 chemical competent cells with 2 ul of the purified reaction mix and plated the bacteria on the appropriate antibiotic LB media (Amp or Kan). We picked two to four colonies and verified the plasmids by restriction digest and Sanger sequencing.

**Online PATC analysis**

The website www.wormbuilder.org/PATC/ was written in R programming language. Its online execution occurs through an Amazon Web Services (AWS) Elastic Computing Cloud (EC2) instance that runs Ubuntu 18.04.3 LTS as the operating system and R version 3.4.4[15] as the working environment. The user interface is created with the "*shiny*" R package version 1.4.0.2[16], and all the graphs are produced either using base R packages or by "*ggplot2*"[17] in conjunction with "*ggvis*"[18] R packages. Please see the accompanying Reporting Summary for detailed version numbers.

Computation of PATC values is performed using a modified PATC algorithm[19], differing from the original by the addition of a "balanced" option that counteracts the effects of long stretches of A or T repetitions[3]. In brief, the algorithm works by assigning a value to each nucleotide in accordance with its separation to AA/TTs motifs; if the value is above the threshold, the base is considered to be in phase of an A/T cluster. The graphic output from the web application highlights the nucleotides in phase by creating an HTML page loaded into the shiny app. Analysis of the PATC periodicity is performed by making a histogram of the distances between AA/TTs motifs followed by a fast Fourier transform with a period of 1 to 20 bp in steps of 0.5.

## Supplementary References


1. Thorvaldsdóttir, H., Robinson, J. T. & Mesirov, J. P. Integrative Genomics Viewer (IGV): high-performance genomics data visualization and exploration. *Brief. Bioinform.* **14**, 178–192 (2013).
2. Stein, L. D. *et al.* The Genome Sequence of Caenorhabditis briggsae: A Platform for Comparative Genomics. *PLOS Biol.* **1**, e45 (2003).
3. Frøkjær-Jensen, C. *et al.* An Abundant Class of Non-coding DNA Can Prevent Stochastic Gene Silencing in the C. elegans Germline. *Cell* **166**, 343–357 (2016).
4. Shaner, N. C. *et al.* Improved monomeric red, orange and yellow fluorescent proteins derived from Discosoma sp. red fluorescent protein. *Nat. Biotechnol.* **22**, 1567–1572 (2004).
5. Gibson, D. One-step enzymatic assembly of DNA molecules up to several hundred kilobases in size. *Protoc. Exch.* (2009) doi:10.1038/nprot.2009.77.
6. Gibson, D. G. *et al.* Enzymatic assembly of DNA molecules up to several hundred kilobases. *Nat. Methods* **6**, 343–345 (2009).
7. Engler, C., Gruetzner, R., Kandzia, R. & Marillonnet, S. Golden Gate Shuffling: A One-Pot DNA Shuffling Method Based on Type IIs Restriction Enzymes. *PLoS ONE* **4**, e5553 (2009).
8. Redemann, S. *et al.* Codon adaptation-based control of protein expression in C. elegans. *Nat. Methods* **8**, 250–252 (2011).
9. Batista, P. J. *et al.* PRG-1 and 21U-RNAs Interact to Form the piRNA Complex Required for Fertility in C. elegans. *Mol. Cell* **31**, 67–78 (2008).
10. Bagijn, M. P. *et al.* Function, targets, and evolution of Caenorhabditis elegans piRNAs. *Science* **337**, 574–578 (2012).
11. Lee, H.-C. *et al.* C. elegans piRNAs mediate the genome-wide surveillance of germline transcripts. *Cell* **150**, 78–87 (2012).
12. Lyssenko, N. N., Hanna-Rose, W. & Schlegel, R. A. Cognate putative nuclear localization signal effects strong nuclear localization of a GFP reporter and facilitates gene expression studies in Caenorhabditis elegans. *BioTechniques* **43**, 596, 598, 560 (2007).
13. Frøkjær-Jensen, C. Insertion of PATC-rich C. elegans introns into synthetic transgenes by golden-gate-based cloning. *Protoc. Exch.* (2020) doi:10.21203/rs.3.pex-1253/v1.

14. Engler, C., Kandzia, R. & Marillonnet, S. A one pot, one step, precision cloning method with high throughput capability. *PloS One* **3**, e3647 (2008).

15. R Core Team. R: A language and environment for statistical computing. Foundation for Statistical Computing, Vienna, Austria. https://www.R-project.org/. (2018).

16. Chang, W., Cheng, J., Allaire, J., Xie, Y. & McPherson, J. shiny: Web Application Framework for R. R package. R package version 1.4.0.2. https://CRAN.R-project.org/package=shiny. (2020).

17. Wickham, H. *ggplot2: Elegant Graphics for Data Analysis.* (Springer-Verlag New York, 2016).

18. Chang, W. & Wickham, H. ggvis: Interactive Grammar of Graphics. R package version 0.4.5. https://CRAN.R-project.org/package=ggvis. (2019).

19. Fire, A., Alcazar, R. & Tan, F. Unusual DNA structures associated with germline genetic activity in Caenorhabditis elegans. *Genetics* **173**, 1259–1273 (2006).
