# Supplementary material for: Engineering rules that minimize germline silencing of transgenes in simple extrachromosomal arrays in C. elegans
Source: Nat Commun. 2020 Dec 9;11:6300. doi: 10.1038/s41467-020-19898-0 (PMC7725773; doi:10.1038/s41467-020-19898-0)
Supplement: Supplementary file 2 — Reporting Summary [file 41467_2020_19898_MOESM2_ESM.pdf]

nature research

Corresponding author(s): Christian Frøkjær-Jensen
Last updated by author(s): Oct 30, 2020

Reporting Summary

Nature Research wishes to improve the reproducibility of the work that we publish. This form provides structure for consistency and transparency in reporting. For further information on Nature Research policies, see our Editorial Policies and the Editorial Policy Checklist.

Statistics

For all statistical analyses, confirm that the following items are present in the figure legend, table legend, main text, or Methods section.

n/a	Confirmed	
☐	☒	The exact sample size (*n*) for each experimental group/condition, given as a discrete number and unit of measurement
☐	☒	A statement on whether measurements were taken from distinct samples or whether the same sample was measured repeatedly
☐	☒	The statistical test(s) used AND whether they are one- or two-sided<br>*Only common tests should be described solely by name; describe more complex techniques in the Methods section.*
☒	☐	A description of all covariates tested
☐	☒	A description of any assumptions or corrections, such as tests of normality and adjustment for multiple comparisons
☐	☒	A full description of the statistical parameters including central tendency (e.g. means) or other basic estimates (e.g. regression coefficient) AND variation (e.g. standard deviation) or associated estimates of uncertainty (e.g. confidence intervals)
☐	☒	For null hypothesis testing, the test statistic (e.g. *F*, *t*, *r*) with confidence intervals, effect sizes, degrees of freedom and *P* value noted<br>*Give P values as exact values whenever suitable.*
☒	☐	For Bayesian analysis, information on the choice of priors and Markov chain Monte Carlo settings
☒	☐	For hierarchical and complex designs, identification of the appropriate level for tests and full reporting of outcomes
☒	☐	Estimates of effect sizes (e.g. Cohen's *d*, Pearson's *r*), indicating how they were calculated

Our web collection on statistics for biologists contains articles on many of the points above.

Software and code

Policy information about availability of computer code

Data collection	Genome-wide PATC values used at www.wormbuilder.dev/PATC/ are from a published Supplemental dataset (Data S2) in Frøkjær-Jensen et al., (2016).
Data analysis	The website www.wormbuilder.dev/PATC/ was written in R programming language, javascript and custom html code. Its online execution occurs through an Amazon Web Services (AWS) Elastic Computing Cloud (EC2) instance that runs Ubuntu 18.04.3 LTS as the operating system system and R version 3.6.3 (R Core Team, 2018) as the working environment. The user interface is created with the “shiny” R package version 1.4.0.2 (Chang et al., 2020) graphics are produced either using base R packages or by ggplot2 version 3.3.0 (Wickham, 2016) in conjunction with ggvis version 0.4.5 (Chang and Wickham, 2019) R packages, and the web browser is produced by igv.js version 2.5.5 (Robinson et al., 2017). The following additional libraries are used to run the app: shinythemes v1.1.2, DT v0.13, shinyWidgets v0.5.1, Cairo v1.5.12, and Biostrings v2.54.0. PATC analysis are performed using the "balanced" PATC algorithm version 05211Balanced (Frøkjær-Jensen et al., 2016) and R base functions. The source code can be obtained at: https://github.com/AmhedVargas/PATC_2_0<br>Graphs and statistical analysis was performed using GraphPad Prism version 8.4.3 (471).<br>Standard DNA editing, three-fragment Gateway assembly, and Golden Gate reactions were performed in silico using ApE (A plasmid Editor) version 2.0.61. ApE is freely available from https://jorgensen.biology.utah.edu/wayned/ape/.

For manuscripts utilizing custom algorithms or software that are central to the research but not yet described in published literature, software must be made available to editors and reviewers. We strongly encourage code deposition in a community repository (e.g. GitHub). See the Nature Research guidelines for submitting code & software for further information.

Data

Policy information about availability of data

All manuscripts must include a data availability statement. This statement should provide the following information, where applicable:

- Accession codes, unique identifiers, or web links for publicly available datasets
- A list of figures that have associated raw data
- A description of any restrictions on data availability

The authors declare that the data supporting the findings of this study are available within the paper and its supplementary information files. Source data for figures [2-7] and Supplementary figures [2-12] are provided with the paper in the accompanying Source Data file.
Gene annotations were gathered from version WS278 from www.wormbase.org.

Field-specific reporting

Please select the one below that is the best fit for your research. If you are not sure, read the appropriate sections before making your selection.

☒ Life sciences ☐ Behavioural & social sciences ☐ Ecological, evolutionary & environmental sciences

For a reference copy of the document with all sections, see nature.com/documents/nr-reporting-summary-flat.pdf

Life sciences study design

All studies must disclose on these points even when the disclosure is negative.

Sample size	Every independently generated transgenic animal (each from a different injected animal) was defined as a single sample. 5-10 independent samples were analyzed per condition based on the variability in fluorescence expression observed in Frøkjær-Jensen et al. (2016).
Data exclusions	No data were excluded from this study.
Replication	To ensure the reproducibility of experiments, several of the most important injections were repeated on (1) different days, and (2) by remaking the injection mixture (see Supplementary Figure 2 for data reproducibility). Results were independently replicated two or three times. All replication attempts were successful.
Randomization	No randomization was necessary as there was no treatment and control groups.
Blinding	Samples were not blinded to the experimenter because different promoters or fluorophores frequently made blinding impractical.

Reporting for specific materials, systems and methods

We require information from authors about some types of materials, experimental systems and methods used in many studies. Here, indicate whether each material, system or method listed is relevant to your study. If you are not sure if a list item applies to your research, read the appropriate section before selecting a response.

Materials & experimental systems

n/a	Involved in the study	
☒	☐	Antibodies
☒	☐	Eukaryotic cell lines
☒	☐	Palaeontology and archaeology
☐	☒	Animals and other organisms
☒	☐	Human research participants
☒	☐	Clinical data
☒	☐	Dual use research of concern

Methods

n/a	Involved in the study	
☒	☐	ChIP-seq
☒	☐	Flow cytometry
☒	☐	MRI-based neuroimaging

Animals and other organisms

Policy information about studies involving animals; ARRIVE guidelines recommended for reporting animal research

Laboratory animals	The standard N2 strain, and mutants derived thereof, were used in this study and are available from the CGC. All experiments were performed on three to five day old hermaphrodites.
Wild animals	No wild animals were used in this study.
Field-collected samples	No field-collected samples were used in this study.

Ethics oversight	These experiments were carried out under the oversight of the Institutional Biosafety and BioEthics Committee (IBEC) at King Abdullah University of Science and Technology under the approved application number 17IBEC34.

Note that full information on the approval of the study protocol must also be provided in the manuscript.
